# Race, the Vaginal Microbiome, and Spontaneous Preterm Birth

**DOI:** 10.1128/msystems.00017-22

**Published:** 2022-05-18

**Authors:** Shan Sun, Myrna G. Serrano, Jennifer M. Fettweis, Patricia Basta, Emma Rosen, Kim Ludwig, Alicia A. Sorgen, Ivory C. Blakley, Michael C. Wu, Nancy Dole, John M. Thorp, Anna Maria Siega-Riz, Gregory A. Buck, Anthony A. Fodor, Stephanie M. Engel

**Affiliations:** a Department of Bioinformatics and Genomics, University of North Carolina at Charlotte, Charlotte, North Carolina, USA; b Department of Microbiology and Immunology and the Center for Microbiome Engineering and Data Analysis, Virginia Commonwealth University, Richmond, Virginia, USA; c Department of Epidemiology, Gillings School of Global Public Health, University of North Carolina at Chapel Hill, Chapel Hill, North Carolina, USA; d Public Health Sciences Division, Fred Hutchinson Cancer Research Center, Seattle, Washington, USA; e Carolina Population Center, University of North Carolina at Chapel Hill, Chapel Hill, North Carolina, USA; f Department of Obstetrics and Gynaecology, School of Medicine, University of North Carolina at Chapel Hill, Chapel Hill, North Carolina, USA; g School of Public Health & Health Sciences, University of Massachusetts Amherst, Amherst, Massachusetts, USA; Duke University

**Keywords:** vaginal microbiome, preterm birth, race, douching

## Abstract

Previous studies have investigated the associations between the vaginal microbiome and preterm birth, with the aim of determining whether differences in community patterns meaningfully alter risk and could therefore be the target of intervention. We report on vaginal microbial analysis of a nested case-control subset of the Pregnancy, Infection, and Nutrition (PIN) Study, including 464 White women (375 term birth and 89 spontaneous preterm birth, sPTB) and 360 Black women (276 term birth and 84 sPTB). We found that the microbiome of Black women has higher alpha-diversity, higher abundance of Lactobacillus iners, and lower abundance of Lactobacillus crispatus. However, among women who douche, there were no significant differences in microbiome by race. The sPTB-associated microbiome exhibited a lower abundance of L. crispatus, while alpha diversity and *L. iners* were not significantly associated with sPTB. For each order of magnitude increase in the normalized relative abundance of L. crispatus, multivariable adjusted odds of sPTB decreased by approximately 20% (odds ratio, 0.81; 95% confidence interval, 0.70, 0.94). When we considered the impact of douching, associations between the microbiome and sPTB were limited to women who do not douche. We also observed strong intercorrelations between a range of maternal factors, including poverty, education, marital status, age, douching, and race, with microbiome effect sizes in the range of 1.8 to 5.2% in univariate models. Therefore, race may simply be a proxy for other socially driven factors that differentiate microbiome community structures. Future work will continue to refine reliable microbial biomarkers for preterm birth across diverse cohorts.

**IMPORTANCE** Approximately 10% of all pregnancies in the United States end in preterm birth, and over 14% of pregnancies end in preterm birth among Black women. Knowledge on the associations between vaginal microbiome and preterm birth is important for understanding the potential cause and assessing risk of preterm birth. Our study is one of the largest studies performed to date to investigate the associations between vaginal microbiome and spontaneous preterm birth (sPTB), with stratified design for Black and White women. We found that the vaginal microbiome was different between Black and White women. The vaginal microbiome was associated with sPTB, and a lower abundance of L. crispatus increased the risk of sPTB independent of racial differences in microbial community structures. Furthermore, we also found that vaginal douching obscured the associations between vaginal microbiome, race, and preterm birth, suggesting that vaginal douching is an important factor to consider in future studies.

## INTRODUCTION

Approximately 10% of all pregnancies in the United States are preterm, over 14% among Black women (1). Intrauterine infection is widely speculated to underlie some portion of preterm deliveries; however, the benefit of prophylactic antibiotic therapy for the prevention of preterm birth (PTB) is not universal and appears to depend at least in part on timing of treatment in pregnancy, route (oral versus intravenous) of antibiotic administration, and clinical presentation (e.g., preterm premature rupture of membranes versus intact membranes) (2–5), which may reflect etiologic heterogeneity among preterm births. One pathway through which pathogenic microorganisms may gain access to the amniotic cavity is by ascending from the vagina and the cervix (6).

The vaginal microbiome plays an important role in the health of the female reproductive tract, and a *Lactobacillus* species-dominated microbiome has been considered important to maintain a healthy state by producing lactic acid and lowering the vaginal pH. In contrast, an increase of bacterial diversity of non-*Lactobacillus* species in the vaginal microbiome, as in bacterial vaginosis (BV), is reported to be an independent risk factor for sexually transmitted infections (STI), PTB, and pelvic inflammatory disease (7, 8). However, there are diverse species of *Lactobacillus* present in the vagina, and these species may produce various levels of lactic acid and have different tolerances for anaerobic members of the microbial community (9). Moreover, some relatively common vaginal microbiome profiles contain few *Lactobacillus* spp. (10, 11). Vaginal microbiomes lacking *Lactobacillus* species of any type tend to have higher pH, more taxon diversity, and often prominently represent organisms that make up the BV diagnosis by Nugent score (7, 11, 12). This profile, which is associated with adverse outcomes, tends to occur more frequently in Black women (11–13). Furthermore, vaginal douching, the practice of intravaginal cleansing with a liquid solution, is more often practiced by Black women (14) and associated with BV (15, 16), although whether BV leads to douching or the reverse is uncertain.

Several studies have attempted to determine whether associations of the vaginal microbiome with PTB differed by race (13, 17–22). However, to date, the majority of these studies have been hampered by insufficiently large sample sizes in all of the included racial/ethnic subgroups. In general, L. crispatus is often reported to be associated with lower risk of PTB, while the taxa related to higher risk of PTB are less consistent between studies and possibly driven by the different racial composition of recruited study participants. Differences in the associations of taxa or community patterns with PTB by race have been underpowered, and it remains uncertain whether a lack of association in any given subgroup is due to inadequate power or the absence of effect.

The present literature appears to support that Black women often have more diverse vaginal microbial community patterns (11, 12) and that women with higher abundance of L. crispatus tend to have overall lower risk of PTB (17–19), although this is not always statistically significant (20). What is not yet clear, given the known racial disparity of PTB (23, 24), is whether microbial community patterns are associated with PTB independent of racial differences in microbial community structure or whether there are differences in the patterns of association between the vaginal microbiome and PTB by race. Furthermore, vaginal douching, a practice more commonly reported by Black women (14), is often associated with vaginal dysbiosis (15, 25, 26) and thus is a critical factor to consider when examining vaginal microbial patterns and preterm birth. To address these questions, we utilized the Pregnancy, Infection, and Nutrition (PIN) Study, a prospectively enrolled pregnancy cohort of women in central North Carolina in the United States, to investigate the relationship between second trimester vaginal microbial community patterns and PTB, and to examine differences in associations by race, after considering important covariates. This well-characterized cohort of women represents the ideal setting to answer this question, given the rich social, behavioral, demographic, and clinical data assembled that describe in detail known determinants of PTB and the large population of Black and White women (27).

## RESULTS

### Maternal characteristics, vaginal microbiome, and spontaneous preterm birth.

**FIG 1:**
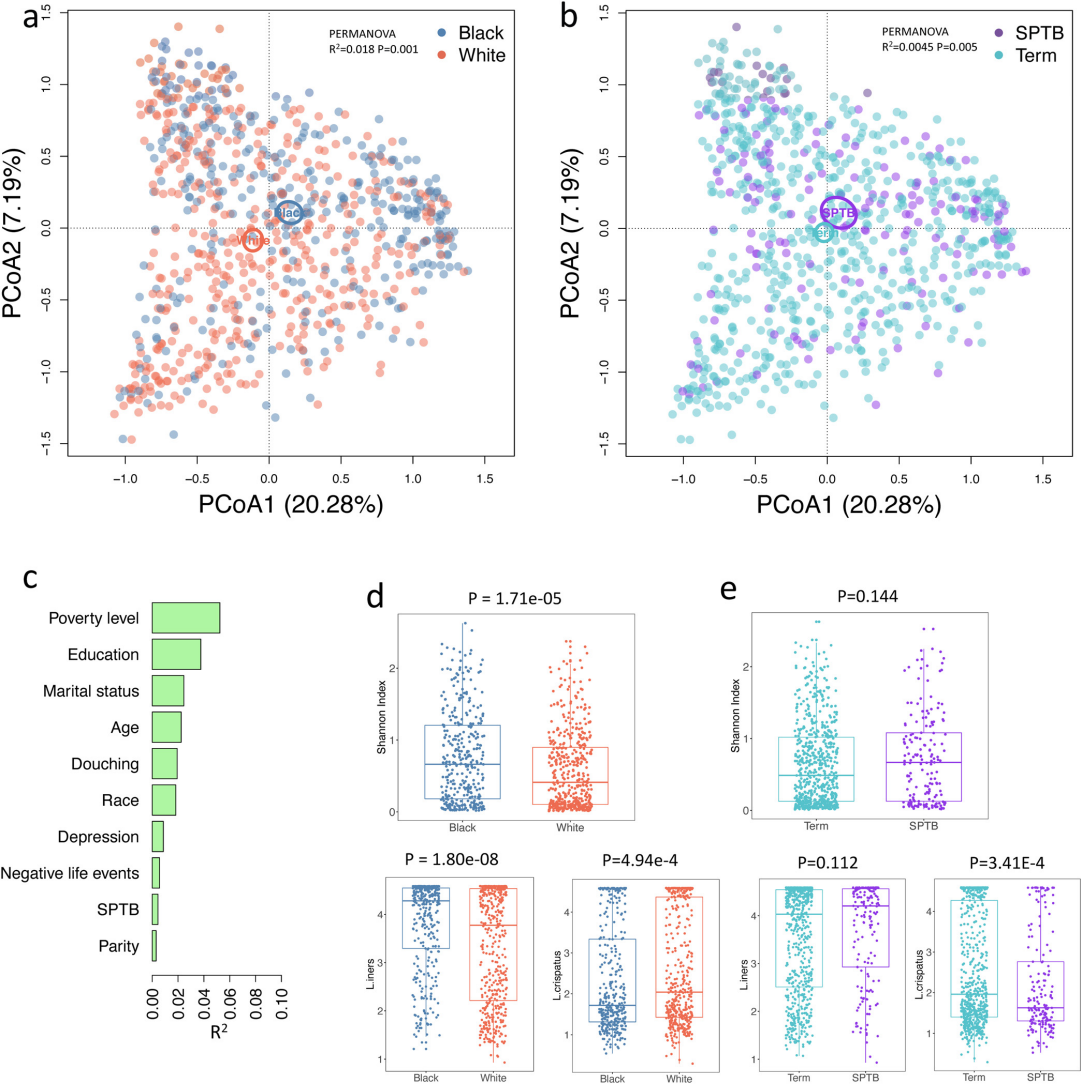
Associations of vaginal microbiome, race, and spontaneous preterm birth (sPTB). PCoA ordination of the vaginal microbiome colored by race (a) and term/sPTB (b). The ellipse indicates a 95% confidence limit of the centroid calculated with the function ordiellipse in R package vegan. (c) The effect sizes (*R*^2^) of host factors with PERMANOVA tests. (d) The associations between race and Shannon diversity for *L. iners* and L. crispatus. (e) The associations between sPTB and Shannon diversity for *L. iners* and L. crispatus. *y* axes show the Shannon diversity and log_10_ normalized relative abundance for *L. iners* and L. crispatus (see Materials and Methods). Significance was determined by Wilcoxon test for Shannon diversity and ALDEx2 for *L. iners* and L. crispatus.

The nested case-control study includes 464 White women (375 term birth and 89 spontaneous PTB, or sPTB) and 360 Black women (276 term birth and 84 sPTB). The characteristics of study participants and their associations with sPTB are shown in Table 1. The complete selection criteria for the analysis are shown in Fig. S1 in the supplemental material. The vaginal microbiome profiles differed significantly by maternal race (permutational multivariate analysis of variance [PERMANOVA], *R*^2^ = 0.018, *P* = 0.001) and sPTB (PERMANOVA, *R*^2^ = 0.0045, *P* = 0.005) (Fig. 1a and b). Principal coordinate analysis (PCoA) ordination of the microbiome showed a separation of the 95% confidence limits of Black and White women (Fig. 1a), with a PERMANOVA *R*^2^ of 1.8%. However, PCoA ordination of the microbiome showed only a modest separation of the 95% confidence limits of sPTB and term birth groups (Fig. 1b), with a relatively small PERMANOVA *R*^2^ (0.45%). A number of maternal features were significantly associated with the vaginal microbiome, including percentage of poverty level, years of education, marital status, age at midpregnancy, douching, self-reported depression, negative life events, and parity (Fig. 1c and Table S1), which are intercorrelated and differentially distributed by maternal race (Fig. S2); therefore, identifying the underlying causal attributes is challenging. Because of the existing literature documenting differences in community patterns across racial and ethnic populations (28), we oriented our results according to maternal self-reported race; however, these patterns likely reflect a complex interplay between social and environmental factors for which race is a marker but not a causal factor. The microbiome of Black women has higher alpha-diversity, higher abundance of *L. iners*, and lower abundance of L. crispatus (Fig. 1d). The spontaneous preterm birth-associated microbiome has lower abundance of L. crispatus, while alpha diversity and *L. iners* were not significantly different (Fig. 1e).

**TABLE 1:** Characteristics of study population, the PIN cohort, 1995 to 2001 (*n* = 824)

Maternal characteristic	White, mean (SD) or *N* (%)			Black, mean (SD) or *N* (%)		
	Term, *N* = 375	sPTB, *N* = 89	*P* value^*a*^	Term, *N* = 276	sPTB *N* = 84	*P* value^*a*^
Maternal age (yr)	27.4 (5.83)	26.9 (7.17)	0.32	24.1 (5.36)	25.4 (5.83)	0.08
Missing	0	0		0	0	
Maternal education (yr)	14.1 (3.32)	13.1 (2.82)	0.02	12.4 (2.00)	12.7 (1.67)	0.11
Missing	0	0		1	0	
Prepregnancy BMI (kg/m^2^)	25.6 (6.93)	25.2 (6.56)	0.79	27.4 (7.99)	27.7 (7.89)	0.66
Missing	7	2		9	6	
Smoking in the 2nd trimester						
Ever	82 (21.9)	30 (33.7)	0.02	25 (9.1)	8 (9.5)	1.00
Never	272 (72.5)	51 (57.3)		215 (77.9)	66 (78.6)	
Missing	21	8		36	10	
Parity						
Nulliparous	168 (44.8)	35 (39.3)	0.34	118 (42.8)	25 (29.8)	0.04
Multiparous	205 (54.7)	54 (60.7)		157 (56.9)	58 (69.0)	
Missing	2	0		1	1	
Marital status						
Single/separated/divorced	117 (31.2)	34 (38.2)	0.21	215 (77.5)	61 (72.6)	0.37
Married	258 (68.8)	55 (61.8)		59 (21.4)	22 (26.2)	
Missing	0	0		3	1	
Maternal household % of poverty level^*c*^	310 (251.2)	268 (227.2)	0.12	135 (110.0)	122 (89.3)	0.69
Missing	32	14		58	13	
Self-reported douching within 12 mo before pregnancy^*b*^						
Yes	77 (20.5)	25 (28.1)	0.02	86 (31.1)	24 (28.6)	0.13
No	173 (46.1)	26 (29.2)		68 (24.6)	10 (11.9)	
Missing^*b*^	125 (33.3)	38 (42.7)		122 (44.2)	50 (59.5)	
CES-D depression symptoms	15.5 (11.3)	15.6 (10.1)	0.67	18.8 (10.4)	19.6 (11.3)	0.80
	68	28		99	45	
Life events inventory, no. of negative life events	3.6 (3.2)	4.2 (3.6)	0.25	3.8 (3.0)	5.2 (4.6)	0.13
	66	28		107	47	
Gestational age at delivery	39.4 (1.3)	34.6 (1.7)	<2.2e−16	39.4 (1.4)	33.8 (2.6)	<2.2e−16
<32 completed wk	0	6 (6.7)		0	14 (16.7)	
32–34 completed wk	0	23 (25.8)		0	24 (28.6)	
35–36 completed wk	0	60 (67.4)		0	46 (54.9)	
≥37 completed wk	375 (100)	0		276 (100)	0	
Preterm clinical presentation						
Preterm labor (PTL)		58 (65.2)			53 (63.1)	
Preterm PROM (PPROM)		31 (34.8)			31 (36.9)	

a For maternal age, years of education, prepregnancy BMI, household poverty, depression symptoms, and negative life events, *P* value was derived from Wilcoxon test. For 2nd trimester smoking, parity, marital status, and douching, *P* value derives from Fisher’s exact test.

b Douching was added to the questionnaire in August 1997, which meant that subjects enrolled prior to this date did not have their douching behavior queried.

c Based on 1996 census.

10.1128/msystems.00017-22.1 FIG S1 Complete selection criteria for the current analysis. Download FIG S1, PDF file, 0.1 MB. Copyright © 2022 Sun et al. 2022 Sun et al. https://creativecommons.org/licenses/by/4.0/ This content is distributed under the terms of the Creative Commons Attribution 4.0 International license.

10.1128/msystems.00017-22.2 FIG S2 Associations between host factors, race and douching groups. Download FIG S2, PDF file, 0.1 MB. Copyright © 2022 Sun et al. 2022 Sun et al. https://creativecommons.org/licenses/by/4.0/ This content is distributed under the terms of the Creative Commons Attribution 4.0 International license.

10.1128/msystems.00017-22.7 TABLE S1 PERMANOVA tests of microbiome and host factors. Download Table S1, DOCX file, 0.01 MB. Copyright © 2022 Sun et al. 2022 Sun et al. https://creativecommons.org/licenses/by/4.0/ This content is distributed under the terms of the Creative Commons Attribution 4.0 International license.

To model these differences with adjustment for covariates, we next calculated multivariable adjusted odds ratios and examined the relative abundance of L. crispatus, *L. iners*, and alpha diversity in relation to sPTB overall and within Black and White women separately via logistic regression (Table 2). Only L. crispatus showed significant associations in White women and in the combined samples. Our examination of interaction terms between L. crispatus and race, however, detected no significant heterogeneity in the associations with sPTB by race (*P* > 0.05). Overall, in the combined samples, each order of magnitude increase in the normalized abundance of L. crispatus was associated with an approximately 20% reduced odds of sPTB (odds ratio [OR] = 0.81; 95% confidence interval [CI], 0.70, 0.94), after accounting for race, maternal education, maternal prepregnancy BMI, and smoking during pregnancy. Relative abundance of *L. iners* and alpha diversity were not significantly associated with sPTB overall or within Black or White women separately. The odds ratios of covariates in the models are shown in Table S2. The odds ratios of univariate models without multivariable adjustment are shown in Table S3.

**TABLE 2:** Multivariable adjusted odds of sPTB associated with L. crispatus, *L. iners*, alpha diversity, and vagitype

Parameter	Overall,^*a*^ OR (95% CI)	Black women,^*b*^ OR (95% CI)	White women,^*b*^ OR (95% CI)	*P* value for test of heterogeneity by race
Log_10_ relative abundance				
L. crispatus	0.81 (0.70, 0.94)	0.85 (0.68, 1.06)	0.80 (0.65, 0.97)	0.66
*L. iners*	1.06 (0.89, 1.27)	1.02 (0.76, 1.36)	1.06 (0.84, 1.34)	0.84
Alpha diversity	1.20 (0.88, 1.64)	1.30 (0.84, 2.02)	1.13 (0.73, 1.77)	0.69
Vagitype				
L. crispatus	0.59 (0.34, 1.01)	0.58 (0.24, 1.42)	0.59 (0.29, 1.19)	0.99
*L. iners*	Ref	Ref	Ref	
Lacto_other	1.52 (0.85, 2.71)	2.78 (1.03, 7.54)	1.15 (0.55, 2.39)	0.16
Others	1.47 (0.91, 2.36)	1.95 (1.00, 3.78)	1.14 (0.57, 2.30)	0.31

a Adjustment set includes race, maternal education, maternal prepregnancy BMI, and smoking during second trimester. Ref, reference value.

b Adjustment set includes maternal education, maternal prepregnancy BMI, and smoking during second trimester.

10.1128/msystems.00017-22.8 TABLE S2 Multivariable adjusted odds of spontaneous preterm birth associated with the covariates included in the models in Table 2. Download Table S2, DOCX file, 0.01 MB. Copyright © 2022 Sun et al. 2022 Sun et al. https://creativecommons.org/licenses/by/4.0/ This content is distributed under the terms of the Creative Commons Attribution 4.0 International license.

10.1128/msystems.00017-22.9 TABLE S3 Associations of spontaneous preterm birth with L. crispatus, *L. iners*, and alpha diversity in univariate models. Download Table S3, DOCX file, 0.01 MB. Copyright © 2022 Sun et al. 2022 Sun et al. https://creativecommons.org/licenses/by/4.0/ This content is distributed under the terms of the Creative Commons Attribution 4.0 International license.

### Vagitypes, spontaneous preterm birth, and race.

**FIG 2:**
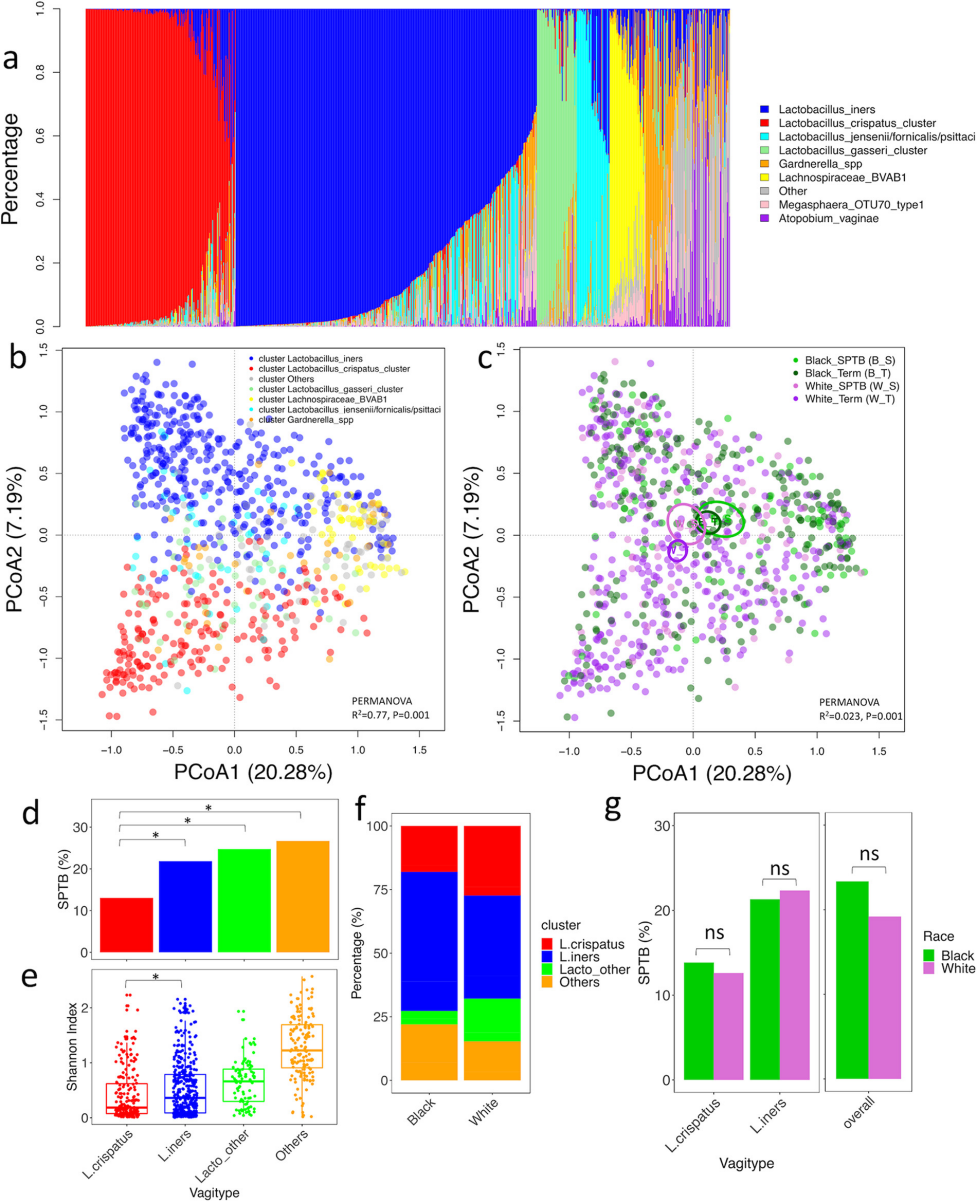
Vagitypes and their associations with spontaneous preterm birth. (a) The vaginal microbiome of study participants showed discrete taxonomic composition that can be classified into vagitypes. (b) The PCoA ordination of the vaginal microbiome colored by vagitypes. (c) The PCoA ordination of the vaginal microbiome colored by race and term/spontaneous preterm birth (sPTB). (d) The percentage of sPTB in each of the vagitypes. Significance was determined with Fisher’s exact test. (e) Shannon diversity of the vagitypes. The Shannon diversity of L. crispatus vagitype was significantly (*P* < 0.05) lower than that of *L. iners* vagitype, estimated with Wilcoxon test. (f) Vagitype composition of Black and White women. (g) The percentage of sPTB associated with L. crispatus and *L. iners* vagitypes in Black and White women. Significance was determined with Fisher’s exact test.

Previous literature has suggested that abstracting microbial community composition into vagitypes is a useful tool for understanding the structure of the vaginal microbial community (20, 28). Taxonomic composition in our study showed that most of the samples were dominated by a single taxon, with prevalent species including *L. iners*, L. crispatus, L. gasseri, L. jensenii*/fornicalis/psittaci*, *Lachnospiraceae* member BVAB1 (BVAB1 has been named provisionally “*Candidatus* Lachnocurva vaginae”) and *Gardnerella* spp. (Fig. 2a; see also Fig. S3 for these data stratified by race, sPTB, and douching). Among these vagitypes, the cluster dominated by *L. iners* has the highest prevalence (46.7%) across all the samples, followed by L. crispatus (23.3%), L. gasseri (6.2%), Lachnospiraceae_BVAB1 (5.6%), and L. jensenii*/fornicalis/psittaci* (5.1%). Because of the discrete community structures of the vaginal microbiome, we classified the microbiome into vagitypes based on the dominant species with relative abundance of >30% by following previously reported methods (17). PCoA ordination of the microbiome colored by these vagitypes showed that different vagitypes generally formed distinguishable clusters, especially for those dominated by *L. iners* and L. crispatus (Fig. 2b). As expected, taxonomic compositions were highly associated with the vagitypes classified (PERMANOVA with 999 permutations, *R*^2^ = 0.77, *P* = 0.001). The same PCoA ordination but colored by race and sPTB showed that the microbiomes of White term-birth women clustered separately from other samples (Fig. 2c), and a PERMANOVA test with these 4 groups (White term, White sPTB, Black term, and Black sPTB) considered separate groups was significant (999 permutations, *R*^2^ = 0.023, *P* = 0.001).

10.1128/msystems.00017-22.3 FIG S3 Taxonomic composition of the vaginal microbiome of study participants stratified based on race, pregnancy outcome, and douching behavior. Download FIG S3, PDF file, 0.2 MB. Copyright © 2022 Sun et al. 2022 Sun et al. https://creativecommons.org/licenses/by/4.0/ This content is distributed under the terms of the Creative Commons Attribution 4.0 International license.

To determine whether the vagitypes were associated with sPTB, we calculated and compared the percentage of sPTB in each vagitype (Fig. 2d). In this analysis, the vagitypes dominated by non-*Lactobacillus* were grouped as Others. The *Lactobacillus* vagitypes except *L. iners* and L. crispatus were grouped as Lacto_other to simplify the model and for sample size considerations. We found that the percentage of spontaneous preterm birth cases with L. crispatus vagitype was significantly lower than that of the other three types, with the spontaneous preterm birth percentages for *L. iners*, Lacto_other, and Others being 1.69, 1.92, and 2 times, respectively, that for L. crispatus (Fisher’s exact tests, *P* = 0.013, 0.019, and 0.0021) (Fig. 2d). Because this study oversampled the underlying cohort for preterm cases, the percentage of preterm birth in this study does not reflect the underlying risk in the population. The Shannon diversity of L. crispatus cluster was significantly lower than that of the other three vagitypes (Fig. 2e).

We next analyzed whether the percentages of vagitypes were different between Black and White women and found Black women had a higher percentage of the *L. iners* and Others vagitype (Fisher’s exact test, *P* = 5.90E−5 and 0.018) and lower percentages of L. crispatus and Lacto_other vagitypes compared to White women (Fisher’s exact test, *P* = 0.0021 and 1.86E−7) (Fig. 2f). We also calculated the multivariable adjusted odds ratios of sPTB, comparing women with the L. crispatus, Lacto_other, and Others vagitypes with women with the more common *L. iners* vagitype. We found no significant heterogeneity in associations by race and that, overall, women with the L. crispatus vagitype had about a 40% reduced odds of sPTB compared to women with the *L. iners* vagitype, although this fell just short of statistical significance (OR = 0.59; 95% CI, 0.34, 1.01). Similarly, women with Lacto_other vagitype and with Others vagitype both had about 50% increased odds of sPTB compared to the *L. iners* vagitype (Lacto_other, OR = 1.52; 95% CI, 0.85, 2.71; Others, OR = 1.47; 95% CI, 0.91, 2.36) (Table 2), but this was also not significant.

We next examined whether the percentages of sPTB cases were different between Black and White women with the same vagitype. In this subset of the PIN cohort, the percentages of Black and White women who had sPTB are not significantly different (Fisher’s exact test, *P* = 0.17) (Fig. 2g). Additionally, there were no significant differences between the percentages of sPTBs in Black and White women with L. crispatus or *L. iners* vagitypes, respectively (Fisher’s exact test, L. crispatus, *P* = 0.82; Fisher’s exact test, *L. iners*, *P* = 0.90). We have verified the results of vagitypes with a different classification method (VALENCIA, for VAginaL community state typE Nearest CentroId classifier) to ensure the results were not dependent on the classification method used (Fig. S4). A power simulation (see the supplemental material) suggested that an analysis of sPTB associated with community state types (CSTs) would lack power for CST groups other than CST I and CST III because of the small number of women with these CSTs. We therefore did not look for association in these samples. Overall, these results with vagitypes are consistent with the analyses in Fig. 1 that shows the higher abundance of L. crispatus associated with reduced sPTB risk.

10.1128/msystems.00017-22.4 FIG S4 Community state types (CSTs) and their associations with preterm birth. (a) The vaginal microbiome of study participants classified to CSTs. (b) The PCoA ordination of the vaginal microbiome colored by CSTs. (c) The PCoA ordination of the vaginal microbiome colored by race and term/spontaneous preterm birth (sPTB). (d) The percentage of sPTB in each of the CSTs. Significance was determined with Fisher’s exact test. (d) Shannon diversity of the CSTs. The Shannon diversity of CST I (dominated by L. crispatus) was significantly lower than that of CST III (dominated by *L. iners*), estimated with Wilcoxon test. (f) CST composition of Black and White women. (g) The percentage of sPTB associated with CST I and CST III in Black and White women. Significance was determined with Fisher’s exact test. Download FIG S4, PDF file, 0.6 MB. Copyright © 2022 Sun et al. 2022 Sun et al. https://creativecommons.org/licenses/by/4.0/ This content is distributed under the terms of the Creative Commons Attribution 4.0 International license.

### Microbiome, douching, and spontaneous preterm birth.

**FIG 3:**
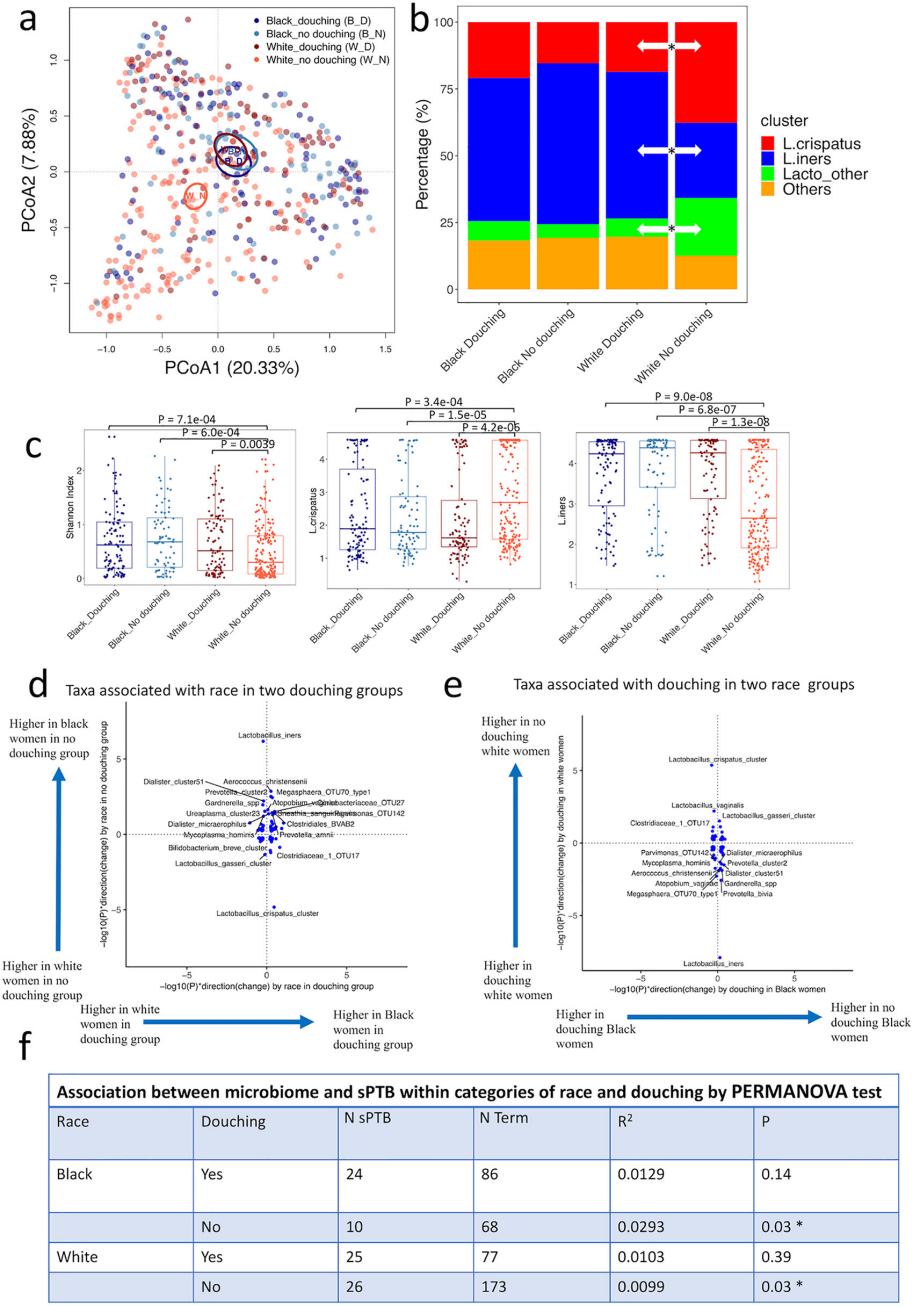
Microbial variation by race and douching and its interference with microbial signatures associated with spontaneous preterm birth (sPTB). (a) PCoA ordination colored by race and douching for samples (*n* = 489) with douching information available. (b) Vagitype composition of douching and nondouching Black and White women. Significance was determined with Fisher’s exact test. (c) Shannon diversity for L. crispatus and *L. iners* abundance by race and douching groups. The Shannon diversity for L. crispatus and *L. iners* abundance was significantly different for no douching White women were compared to the other three groups. Shannon diversity was analyzed with Wilcoxon test and the taxa abundance was analyzed with ALDEx2. (d) Comparison of microbial species associated with race in douching and no douching participants. The *x* axis is −log_10_(*P*) of the associations between each taxon and race with ALDEx2 in douching group, multiplied by the direction of changes, while the *y* axis is in no douching group. (e) Comparison of microbial species associated with douching in Black and White participants. The *x* axis is −log_10_(*P*) of the associations between each taxon and douching with Wilcoxon test in Black women, multiplied by the direction of changes, while the *y* axis describes White women. (f) PERMANOVA tests of the associations between microbiome and term/sPTB birth in each of the race and douching groups.

Douching information was added to the PIN questionnaire in August 1997 and thus was only available on a subset of the enrolled population (Table 1). The characteristics of study participants with or without douching are shown in Table S4. To better understand the relationship between maternal race, douching, and the vaginal microbiome, we created 4 distinct groups for a subset of participants with douching information (*n* = 489): Black, no douching (B_N; *n* = 78); Black, douching (B_D; *n* = 110); White, no douching (W_N; *n* = 199); and White, douching (W_D; *n* = 102). PCoA ordination showed that the White nondouching group formed a separate cluster from the other three groups (Fig. 3a). This was supported by the PERMANOVA tests that indicated that the White nondouching group is significantly different from the Black nondouching group (*R*^2^ = 0.0284, *P* = 0.001), while the White and Black douching groups were not significantly different from each other (*P* = 0.40). This is consistent with the vagitype composition of these four groups, with nondouching White women associated with higher percentage of L. crispatus and Lacto_other and lower percentage of *L. iners* vagitype compared to nondouching Black women (Fisher’s exact tests, *P* = 0.00028, 0.00061, and 1.04E−6), while the Black and White women who douche were not significantly different from one another (Fisher’s exact tests, *P* = 0.73, 1.00, and 0.89) (Fig. 3b). While douching was associated with a significant difference in the relative proportion of vagitypes L. crispatus, *L. iners*, and Lacto_other among White women (Fisher’s exact tests, *P* = 6.4E−4, 8.8E−6 and 9.4E−4), it was not associated with a significant difference in the relative proportion of vagitypes L. crispatus, *L. iners*, and Lacto_other among Black women (Fisher’s exact tests, *P* = 0.45, 0.38, and 0.76) (Fig. 3b). Additionally, the Shannon diversity and the abundance of *L. iners* and L. crispatus showed that the White nondouching group was significantly different from the other three groups (Wilcoxon test for Shannon diversity and ALDEx2 for *L. iners* and L. crispatus, *P* < 0.05) (Fig. 3c). We analyzed the individual species associated with race and douching with pairwise ALDEx2. After adjusting for multiple hypothesis testing, there were four taxa (L. crispatus, *L. iners*, Aerococcus christensenii, and *Prevotella* cluster2) that showed significant associations with race in the nondouching participants, but none were significant by race in the douching group (Fig. 3d). Likewise, there are three taxa (L. crispatus, *L. iners*, and Prevotella bivia) associated with douching in White participants but no taxa associated with douching in Black participants (Fig. 3e). Taken together, these data show that the association of douching and the microbiome was much stronger for White women, while race was significantly associated with the variation of the vaginal microbiome only for nondouching participants.

10.1128/msystems.00017-22.10 TABLE S4 Characteristics of study participants with or without douching behavior within 12 months before pregnancy (*n* = 489). Download Table S4, DOCX file, 0.01 MB. Copyright © 2022 Sun et al. 2022 Sun et al. https://creativecommons.org/licenses/by/4.0/ This content is distributed under the terms of the Creative Commons Attribution 4.0 International license.

We next examined the relationship of sPTB and the microbiome in the four groups. With PERMANOVA tests, the vaginal microbiome was significantly associated with sPTB only in the nondouching participants for both race groups (Black, *P* = 0.030; White, *P* = 0.034), while this association was not significant for the douching participants (Black, *P* = 0.14; White, *P* = 0.39) (Fig. 3f). Moreover, compared to the PERMANOVA test for pregnancy outcomes without stratification of race and douching, the effect sizes (PERMANOVA *R*^2^) here are much increased, from 0.45% to 3% in Black women and 1% in White women.

## DISCUSSION

In this large, prospective pregnancy cohort, we analyzed the association between the midpregnancy vaginal microbiome, race, and sPTB. We found that vaginal microbiomes were significantly associated with sPTB, race, douching, and other maternal factors. Many of these maternal factors, like poverty, education, marital status, age, douching, and race, have stronger associations with the vaginal microbiome than the vaginal microbiome has with sPTB (Fig. 1c). Consistent with previous studies (11, 28), we found that the vaginal microbiomes of Black and White women were significantly different, with higher alpha diversity, higher abundance of *L. iners*, and lower abundance of L. crispatus for Black women. The microbial difference between sPTB and term controls is mainly driven by a higher L. crispatus abundance in term controls, similar to previous reports (17, 18). Because of the strong intercorrelations between maternal factors such as race, poverty, education, marital status, and douching (Fig. S2), we stratified the data set by race and douching with the aim of uncovering potentially stronger sPTB microbial signatures that are independent of race and douching.

With the community state types assigned based on the most abundant taxon, the sPTB risk associated with the L. crispatus-dominated community state is about 60% of that for the *L. iners*-dominated microbiome (Fig. 2d). The alpha diversity of the L. crispatus-dominated microbiome is significantly lower than that of the *L. iners*-dominated microbiome, indicating that L. crispatus suppresses the colonization and development of a BV-like microbiome while *L. iners* does not. This observation is consistent with previous literature that has found that a vaginal microbiome dominated by *L. iners* also more often shifts toward a diverse community compared to L. crispatus (29). Similarly, it has been shown that *L. iners* enhances the adhesion of *Gardnerella* spp. to cervical epithelial cells, and *Gardnerella* spp. displaced adherent L. crispatus but not *L. iners* from epithelial cells (30). Previous research also suggests that L. crispatus and *Gardnerella* tend to be exclusive members of the microbial community while *L. iners* and *Gardnerella* often show patterns of coexistence (18). This exclusion hypothesis may explain the higher sPTB risk associated with the *L. iners*-dominated microbiome than L. crispatus in our population. In further support of this idea, the sPTB risk associated with the community state “Lacto_other” dominated by other *Lactobacillus* species (mostly L. gasseri and L. jensenii*/fornicalis/psittaci*) is also significantly higher than that of L. crispatus-dominated community state, indicating that these species also are not as protective as L. crispatus.

With a similar number of Black and White participants in our study, we analyzed the associations between the microbiome and sPTB risk in each race separately and found that risk of sPTB associated with L. crispatus and *L. iners* are similar for Black and White women (Fig. 2g). Although at the U.S. population level Black women have substantially higher risk of PTB (23), in the PIN study specifically, Black race is only marginally associated with PTB (OR, 1.3; 95% CI, 1.0, 1.6) (27). Our findings that race does not modify the association between L. crispatus and *L. iners* and sPTB (Fig. 2g) suggest that the disparity in PTB rates at the U.S. population level in part are due to the lower prevalence of L. crispatus-dominated microbiome among Black women. In terms of the impact of taxa beyond L. crispatus and *L. iners*, sample size considerations prevented us from determining whether race can modify the sPTB risk associated with other species. For example, the vagitype “Others” consisted of many BV-related species and had a higher percentage of sPTBs, but the modest number of women in this group relative to the large number of different species represented by the Others group makes attempts to measure the impact of individual species unreliable. Future studies will be needed to further investigate the other species.

Douching is often associated with BV (15, 25, 26), although it is difficult to determine whether douching increases the risk of BV or BV leads to douching, or whether these are correlated but not causally linked phenomena. In a prior investigation within the PIN cohort, douching prior to pregnancy and BV were independently but not jointly associated with increased risk of PTB, i.e., douching did not modify the relationship between BV and preterm birth, and BV did not modify the relationship between douching and PTB (31). However, this prior study did not consider the impact of self-reported race.

In our study, we found that douching in the 12 months before pregnancy played an important role in the structure of the vaginal microbiome during pregnancy among White women in particular. Among women who did not douche, Black and White women had clearly different microbiomes (Fig. 3a and b), and both were associated with sPTB (Fig. 3f). Among women who did douche, however, the microbiomes of Black and White women were similar (Fig. 3a and b) and not associated with sPTB (Fig. 3f). Our findings suggest that had we conducted a study of only women who douche, race would have played no role in differentiating microbiome composition, as all taxa had *P* values close to 1 compared to Black and White women that douche (Fig. 3d, *x* axis). Similarly, had we conducted a study of only Black women, our findings suggest that douching would have played no role differentiating microbiome composition, as when comparing douching and nondouching Black women, all taxa in the microbiomes had *P* values close to 1 (Fig. 3e, *x* axis). The effect displayed by these figures is striking, but the immediate causal implications for the relationships between race, microbiome, sPTB, and douching are unclear. One hypothesis is that douching disrupts the healthier L. crispatus-dominated microbiome, which then shifts to a higher-risk microbiome, ultimately leading to sPTB. However, we see evidence for this hypothesis in White women but not Black women, who do not show substantial shifts in microbiome in relation to douching (Fig. 3b). Another possible explanation for this discrepancy is that a preexisting dysbiotic state causes both douching behavior and sPTB and that this preexisting state is more commonly found in Black women. Future studies with longitudinal vaginal tract sampling, and longitudinal information on douching behavior, will be required to understand why douching behavior is significantly associated with the vaginal microbiome only in White women in our study and why there are no race-related differences in the vaginal microbiomes among women who report douching.

In summary, in this prospective study of midpregnancy microbiome and sPTB in a well-characterized cohort of Black and White women, we found that the vaginal microbiome of Black women was characterized by higher diversity, lower abundance of L. crispatus, and higher abundance of *L. iners*. These differences were obscured once maternal douching behavior was considered; specifically, among women who douche, there were no material differences in microbiome by race. Additionally, we found that women with microbiome dominated by L. crispatus had lower risk of sPTB than those dominated by *L. iners*, and these associations were the same for Black and White women. To our knowledge, this is the first study of the vaginal microbiome and sPTB to consider the impact of douching, and we found that douching is an important factor to consider in future studies.

Our study has a number of limitations. As with all association studies, the observed associations do not necessarily demonstrate or reflect mechanistic causality where differences in the microbial community directly cause risk of preterm birth. It is important to note that while we present differences in microbial community patterns by race that are consistent with the prior literature (11, 12), we observed strong intercorrelations across a number of maternal factors whose effects cannot easily be separated. These intercorrelated factors include race, douching, poverty level, psychosocial stress, education, marital status, and maternal age. In this, as with many other medical research studies, maternally self-classified race only crudely captures complex social determinants of health (32); thus, disparities in microbial community patterns that we observe in relation to race may actually result from factors such as diet, access to high-quality medical care, behaviors like vaginal douching, social support and life experiences, psychosocial stress, and experiences of discrimination. Therefore, factors that may explain differences in vaginal microbial community patterns by race need further investigation and elucidation. In a recent investigation of Black women enrolled at a single study site, Dunlop and colleagues found low levels of education, but not prenatal insurance, marital status, or antibiotic use, was associated with Shannon diversity of the vaginal microbiome (33). We also found that education, and even more so, poverty, accounted for substantial amounts of microbiome variation that were higher than the variations explained by race or douching alone (Fig. 1c). We further found that differences in the microbiome by race were erased among women who douche. In the PIN study, self-reported douching is more commonly reported among Black women, women who are not married at the time of their pregnancy, women with lower levels of education, women with higher prepregnancy BMIs, and those who smoke in the first 6 months of pregnancy (31). Disentangling these correlated factors will require large and diverse populations, which to date do not exist in the literature. In addition, it is also important to note that studies that recruit women from prenatal clinics may not be representative of all pregnancies, thus presenting the opportunity for selection bias. In a previous examination of the PIN cohort, women recruited into the study from prenatal clinics were compared to women who resided in the geographical area of the study (34). Among other features, participants in PIN were more likely to be Black, younger, have lower education, be unmarried, and smoke more cigarettes during pregnancy. While we accounted for these characteristics in our multivariable adjusted models, such differences are important to remain conscious of when interpreting results from clinic-based populations.

Another limitation of our study is that we did not sample longitudinally, and therefore we are unable to evaluate the stability of the association between the microbiome and PTB across gestation. Previous studies have suggested a longitudinal signal with some taxa changing with time in ways that are different in different populations (17). The relationship between time, sPTB, and the microbiome is an important question that should be addressed in future studies. Our study also did not explicitly consider the impact of biomolecules such as defensins. It is likely that interindividual differences in host response, such as through promotion of inflammatory cytokine or chemokine production (35, 36), or interactions with vaginal defensins (20), influence the overall association of vaginal microbial community patterns and sPTB. Previous research within PIN has found that vaginal fluid neutrophil defensin concentration was not strongly associated with PTB (37), but another study found that defensin levels were associated with intermediate BV (38). Much remains unknown as to the biological mechanisms linking the vaginal microbiome and preterm birth, and future studies should consider the extent to which variation in host defense mechanisms plays an important mediating role.

Although our study is one of the largest studies of the associations between the vaginal microbiome and sPTB, it still lacks power for analyzing less abundant microbes and vagitypes and whether the combination of race or other social factors and douching influences the consistency of microbial signatures. A strength of our study is that it demonstrates how not accounting for social and behavioral factors could lead to discrepant results across studies of the vaginal microbiome. For example, our results suggest that two studies with very different percentages of White and Black women would likely report contradictory results in the association of the microbiome with douching. Pooled studies across cohorts with similar metagenomics data may enable a more precise investigation of rare species as well as the association of maternal factors that may explain or modify effects of the vaginal microbiome on sPTB.

## MATERIALS AND METHODS

### Study population.

The PIN study enrolled pregnant women with singleton pregnancies in central North Carolina from August 1995 to February 2001. Women were recruited from prenatal clinics at the University of North Carolina Hospitals, Wake County Human Services, and the Wake Area Health Education Center. Eligibility criteria included gestational age at enrollment between 24 and 29 weeks, ability to communicate in English, age 16 years or older, access to a telephone, and plans to deliver at the recruitment site (39). At enrollment, women provided blood, urine, and genital tract specimens. In the subsequent 2 weeks following enrollment, women completed a telephone interview that collected information on social, demographic, medical, and behavioral risk factors for adverse pregnancy outcomes. In total, 3,163 women were recruited into the PIN study during this period. This study was approved by the Institutional Review Board at UNC Chapel Hill (IRB number 16-2166).

For the current nested case-control study, we selected from the total PIN population all of the 402 women who went on to have a PTB and randomly sampled 799 women from among term births as the control group. The current study was restricted to spontaneous preterm birth and control members who self-classified as Black or White race (*n* = 824), including 375 White women with term birth, 89 with spontaneous preterm birth, 276 Black women with term birth, and 84 with spontaneous preterm birth. Based on our final sample size, the case/control ratio was approximately 1:3.8. While we could have theoretically sampled more controls into our study, there are negligible power gains when adding only controls but no additional cases once the ratio exceeds 4:1 (40–42). Complete selection criteria for the current analysis are described in Fig. S1 in the supplemental material.

### Preterm birth clinical presentation.

Gestational age at delivery was assigned by early ultrasound (completed prior to 22-week gestation) in 90% of the population or last menstrual period date if ultrasound was unavailable (43). The primary study outcome is spontaneous preterm birth. This included births <37 completed weeks of gestation with clinical presentation of preterm labor (PTL) or preterm premature rupture of membranes (PPROM). Term birth was defined as ≥37 completed weeks of gestation. Preterm clinical presentation was determined by obstetrician review and classified as PTL, PPROM, in which membranes ruptured four or more hours before the onset of labor and were medically indicated. For the current study, we combined PTL and PPROM into a single clinical presentation of spontaneous preterm birth (sPTB). Among sPTB cases, gestational ages varied from 26 to 36 completed weeks (Table 1).

### Covariates.

We selected covariates for consideration as predictors of the microbiome or confounders of the microbiome-PTB association based on prior literature (31, 33, 44, 45). These included maternal age, maternal education, marital status, self-reported prepregnancy weight and height to calculate body mass index (BMI), parity, any smoking in the 2nd trimester of pregnancy, maternal household percentage of poverty index based on the 1996 census, douching in the 12 months before pregnancy (added to the questionnaire in August 1997), maternal self-reported depressive symptoms, and the number of negative life events. Maternal depressive symptoms were measured based on the Center for Epidemiological Studies depression scale (CES-D) (46). Negative life events were assessed by a modified life events inventory (LEI) (47). For the purpose of multivariable adjusted analyses, we identified a minimally sufficient subset of adjustment variables based on a directed acyclic graph (DAG) (48) using daggity (49). Our final adjustment set included maternal race, douching, maternal education, BMI, and smoking.

### DNA extraction and sequencing.

Swabs were collected by clinicians between 24 and 29 weeks of gestation from the posterior vaginal apex and stored at −70°C. For the current study, swabs were thawed on ice and processed as previously described (17). In brief, DNA was extracted using the PowerSoil DNA isolation kit (Qiagen), eluted in 100 μL water, and quantified using PicoGreen. Extracted DNA was amplified with molecularly barcoded primers targeting the V1 to V3 hypervariable regions of the bacterial 16S rRNA gene using protocols established in the Vaginal Human Microbiome Project at VCU (12). Samples were multiplexed (384 samples/run) using a sample-specific dual-index strategy and sequenced on Illumina MiSeq sequencers (2 × 300 base paired-end protocol). The paired-end quality-aware raw sequence files were demultiplexed into sample-specific data and merged and quality-filtering using MeFiT (50). Samples with fewer than 1,000 high-quality reads were excluded. The sequences generated can be accessed at NCBI with BioProject ID PRJNA694098. Additional details on swab collection, processing, and quality control can be found in the supplemental material.

### Bioinformatics and statistical analysis approach.

The primary analysis aims of this NIH-funded study were to examine the association of the vaginal microbiome with sPTB overall and within strata of race and to ascertain predictors of vaginal microbial signatures. Our overall statistical plan was to characterize the vaginal microbiomes and build a series of models to attempt to determine which taxa were associated with sPTB in our study. Although we do not include detailed prospective power simulations in the manuscript, our study is one of the largest vaginal microbiome studies so far, and we found many significant associations between the microbiome and maternal factors. Comprehensive 16S rRNA gene-based taxonomic survey of the vaginal microbial profiles yielded a mean count of 43,276 reads/sample with minimum and maximum read counts of 1,824 and 186,784, respectively. Over 99.9% of the high-quality sequencing reads generated overlapping paired-end reads. High-quality sequences were assigned to the species-level taxonomic assignments for vaginal samples using STIRRUPS (51), an analysis platform that employs the USEARCH algorithm (52), combined with a curated vaginal 16S rRNA sequence database that can classify over 95% processed reads to the species level. Paired reads that did not align to the same reference sequence were discarded as chimeras. Analyses with DADA2 and SILVA 132 release were used as alternative pipelines. The relative abundance of microbial species was normalized with multiplying the proportion by the average sequencing depth of all the samples (53) as described below:

(1) $$\mathrm{Normalized}\;\mathrm{abundance}\;\mathrm{of}\;\mathrm{taxon}\;i\;\mathrm{in}\;\mathrm{sample}\;j=\frac{\mathrm{Number}\;\mathrm{of}\;\mathrm{reads}\;\mathrm{classified}\;\mathrm{as}\;\mathrm{taxon}\;i\;\mathrm{in}\;\mathrm{sample}\;j}{\mathrm{Total}\;\mathrm{number}\;\mathrm{of}\;\mathrm{reads}\;\mathrm{in}\;\mathrm{sample}\;j}\times \mathrm{Average}\;\mathrm{number}\;\mathrm{of}\;\mathrm{reads}\;\mathrm{per}\;\mathrm{sample}$$

Because of the variation of sequencing depth across samples, we tested if the sequencing depth were associated with race, sPTB, and douching with Wilcoxon test and found none of the associations were significant (Fig. S5). We also rarefied the samples to the minimum number of reads per sample (1,654) and recalculated the alpha- and beta-diversity and the relative abundance of *L. iners* and L. crispatus to ensure that the results were not an artifact of sequencing depth (Fig. S6).

10.1128/msystems.00017-22.5 FIG S5 Sequencing depth of samples was not associated with race, SPTB, or douching. Download FIG S5, PDF file, 0.2 MB. Copyright © 2022 Sun et al. 2022 Sun et al. https://creativecommons.org/licenses/by/4.0/ This content is distributed under the terms of the Creative Commons Attribution 4.0 International license.

10.1128/msystems.00017-22.6 FIG S6 Associations of race and sPTB with the alpha- and beta-diversity and abundance of *L. iner* and L. crispatus calculated from samples rarefied to the minimum number of reads per sample (1,654). Download FIG S6, PDF file, 0.3 MB. Copyright © 2022 Sun et al. 2022 Sun et al. https://creativecommons.org/licenses/by/4.0/ This content is distributed under the terms of the Creative Commons Attribution 4.0 International license.

The PCoA ordinations were calculated based on the Bray-Curtis dissimilarity between samples with function capscale and visualized with ordiplot in R package vegan. PERMANOVA tests were used to analyze the associations between microbiome and host factors with function adonis in the same package. Shannon index was used to calculate the alpha-diversity of microbial communities (54).

Two methods were applied to determine the vaginal microbial community states or vagitypes based on the taxonomic composition. First, vagitypes were determined by the most abundant species with a relative abundance of >30%, and that species was considered the dominant species of that vagitype. The microbiome was characterized as “no type” when the relative abundance of all species was lower than 30%. We also used the VALENCIA (VAginaL community state typE Nearest CentroId classifier) that is a nearest centroid classification method and analyzed the community state types (CSTs) (55). Over 95% of classifications of the two methods were consistent. Associations between sPTB, vagitypes, and host factors were determined with Fisher’s exact test. The associations between species and sPTB, race, and douching were primarily analyzed with ALDEx2. *P* values were adjusted using the Benjamini-Hochberg method (56) if more than five taxa were tested. To estimate multivariable adjusted associations between alpha diversity, selected taxa, and sPTB, we employed logistic regression using the R function glm, adjusting for covariate sets identified by our DAG. Adjusted odds ratios (OR) and 95% confidence intervals (95% CI) are reported. Missing data were not included in the analysis. Quantitative variables were not categorized.

### Ethics approval and consent to participate.

This study was approved by the Institutional Review Board at UNC Chapel Hill. Informed consent was obtained from all participants.

### Data availability.

The sequences generated can be accessed at NCBI with BioProject ID PRJNA694098. R scripts used in this study and participants’ metadata (race, sPTB, and douching practice) are available at GitHub (https://github.com/ssun6/PINmicrobiome).
