# Supplementary figures and images for: Race, the Vaginal Microbiome, and Spontaneous Preterm Birth
Source: mSystems. 2022 May 18;7(3):e00017-22. doi: 10.1128/msystems.00017-22 (PMC9238383; doi:10.1128/msystems.00017-22)

Pregnancy, Infection and Nutrition Cohort 1995-2003  
(n = 3163)

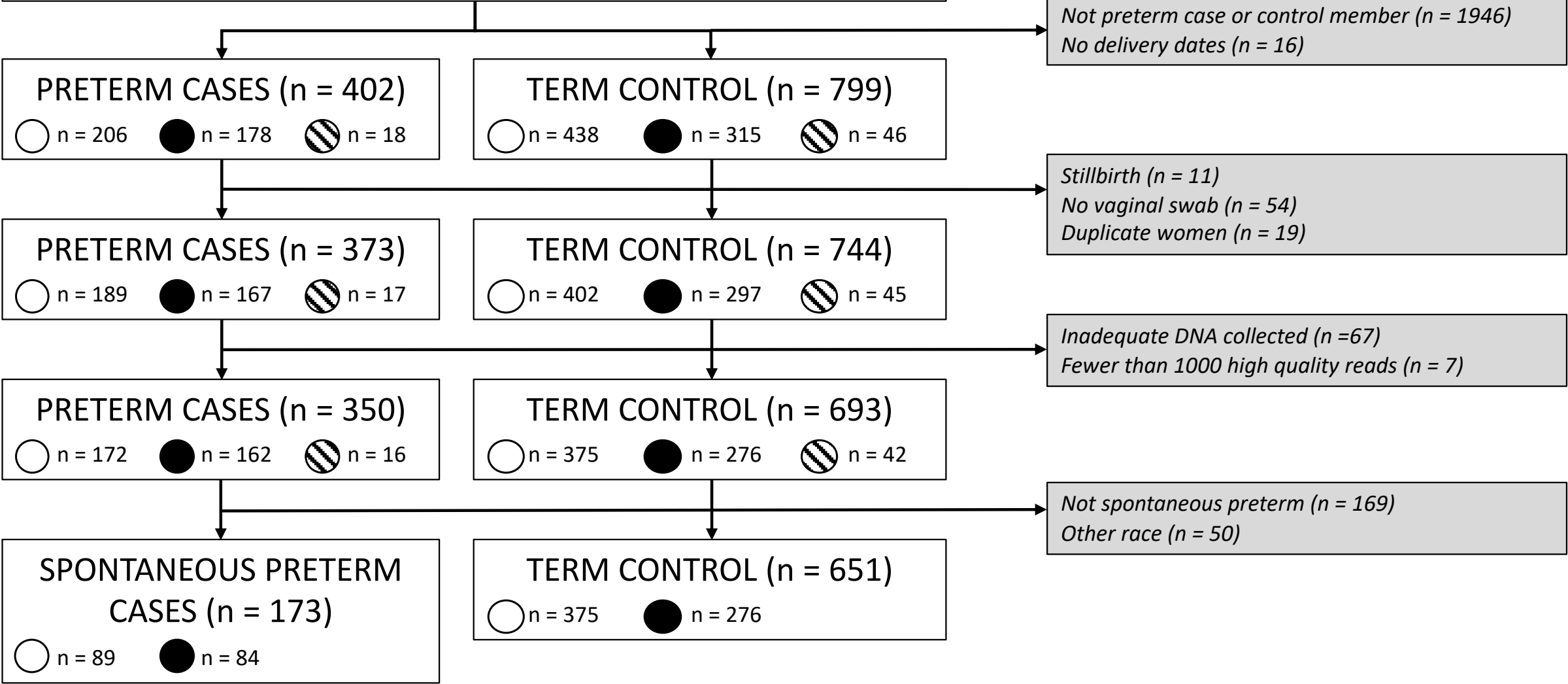

WHITE ○ BLACK ● OTHER ◐

Supplement: FIG S1 [file msystems.00017-22-s0005.pdf]

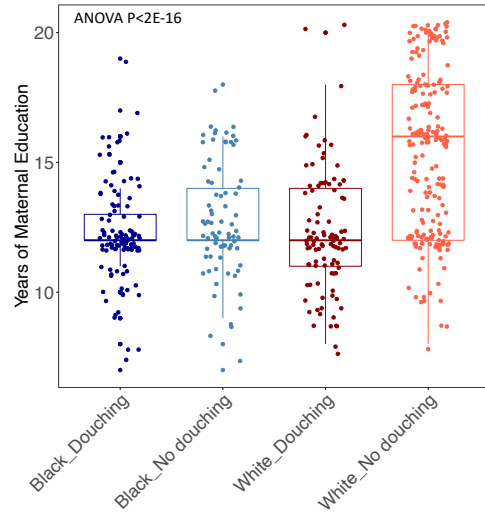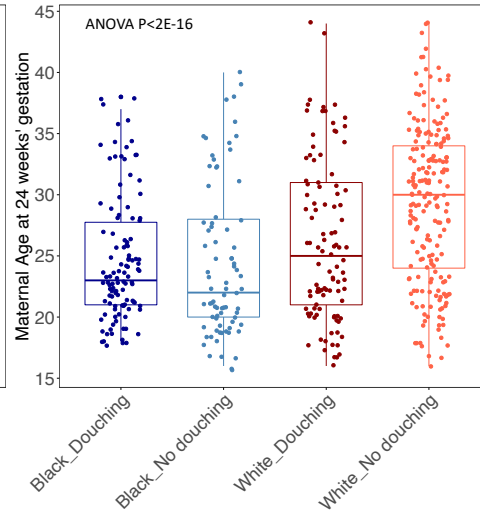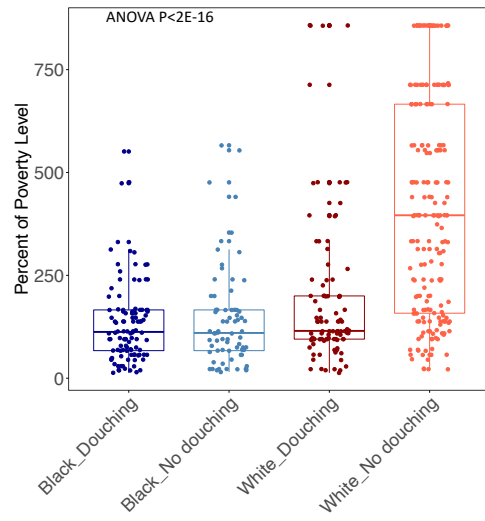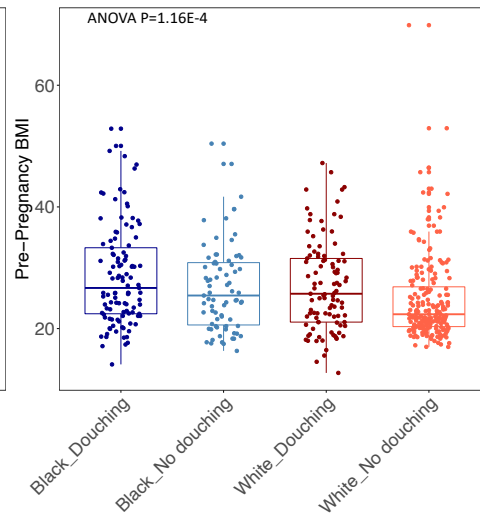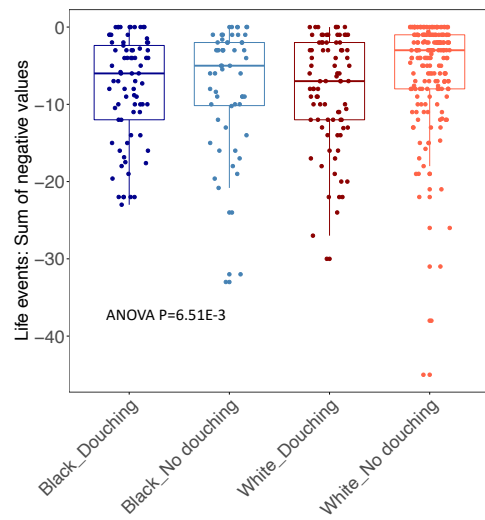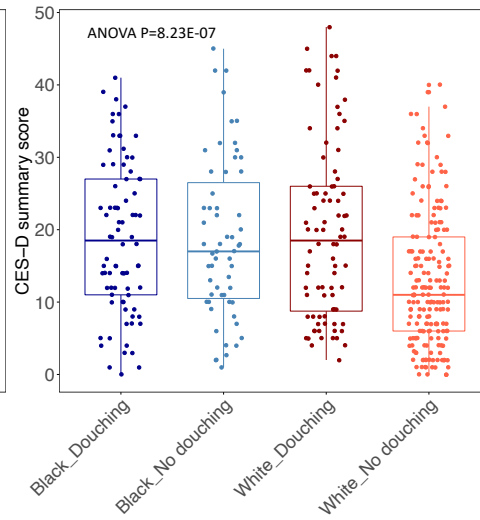

Supplement: FIG S2 [file msystems.00017-22-s0006.pdf]

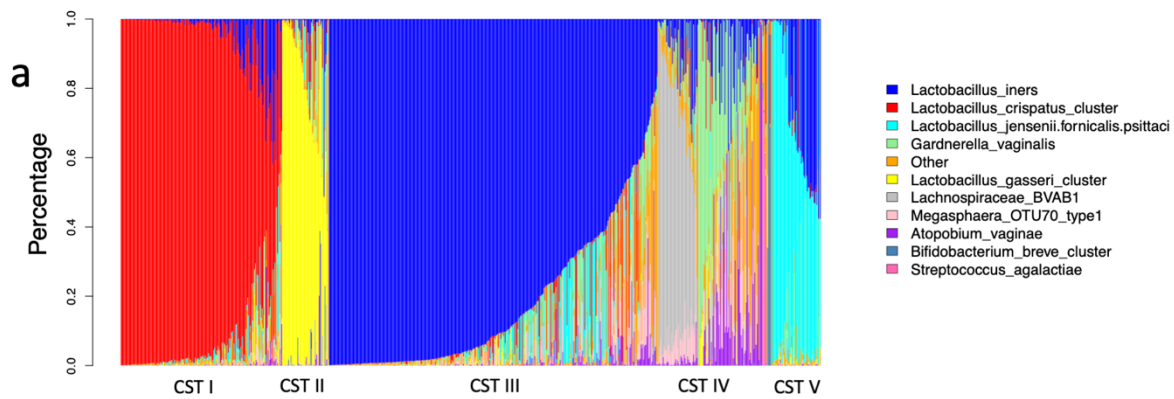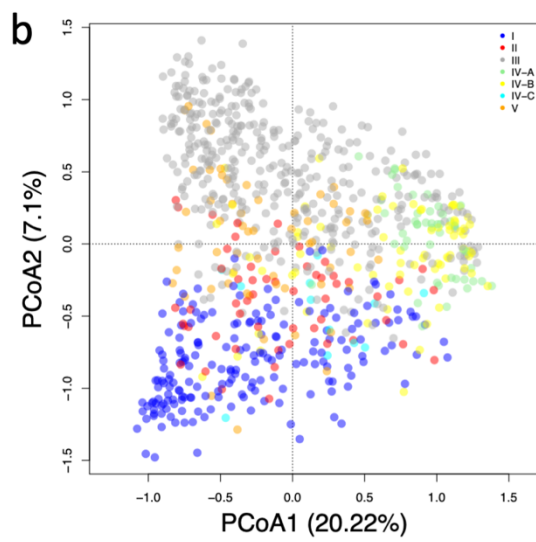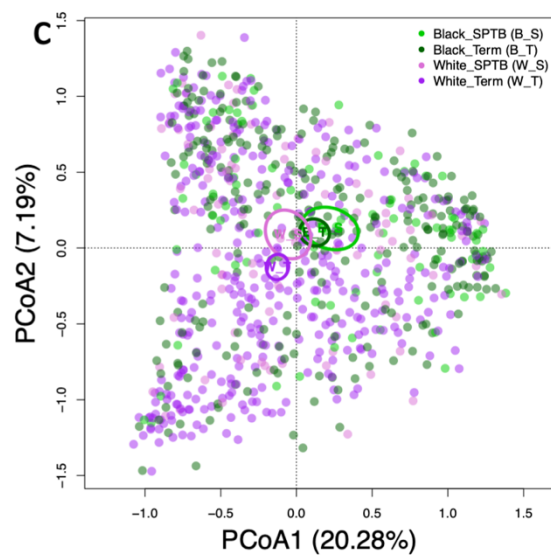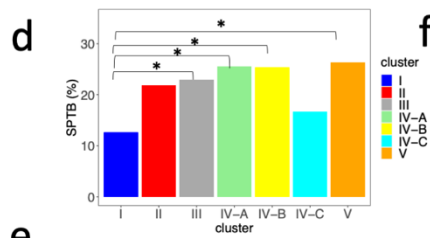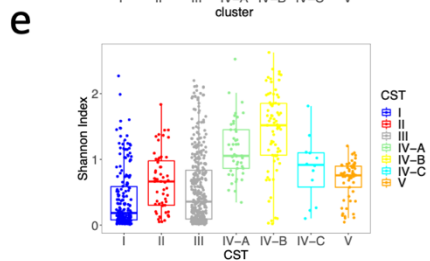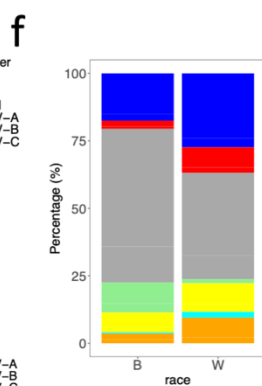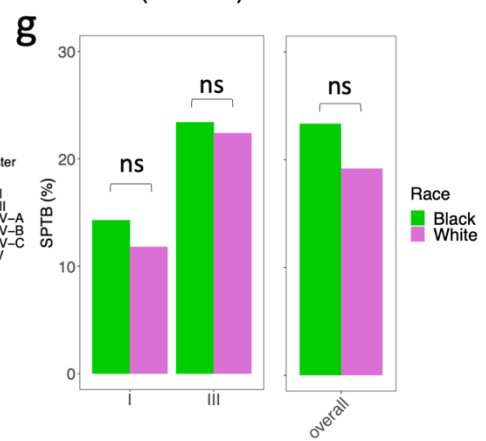

Supplement: FIG S4 [file msystems.00017-22-s0008.pdf]

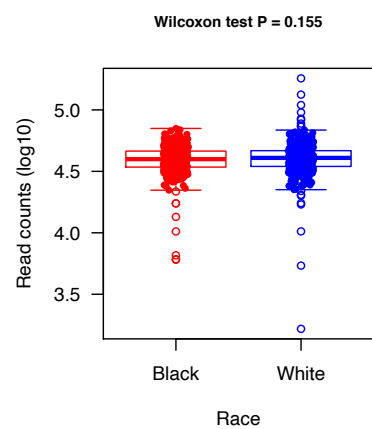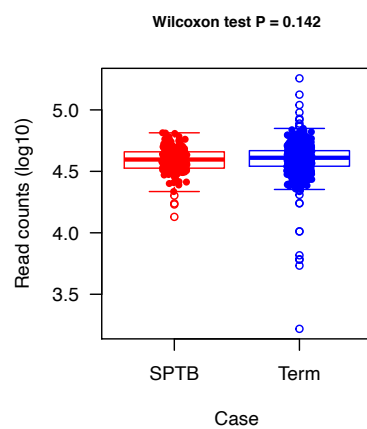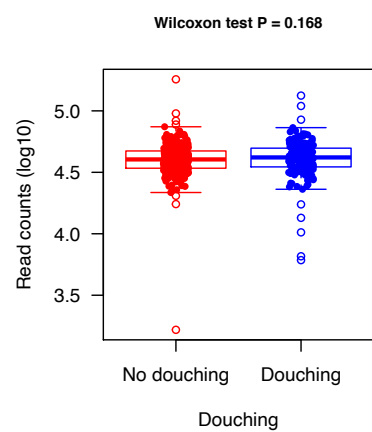

Supplement: FIG S5 [file msystems.00017-22-s0009.pdf]

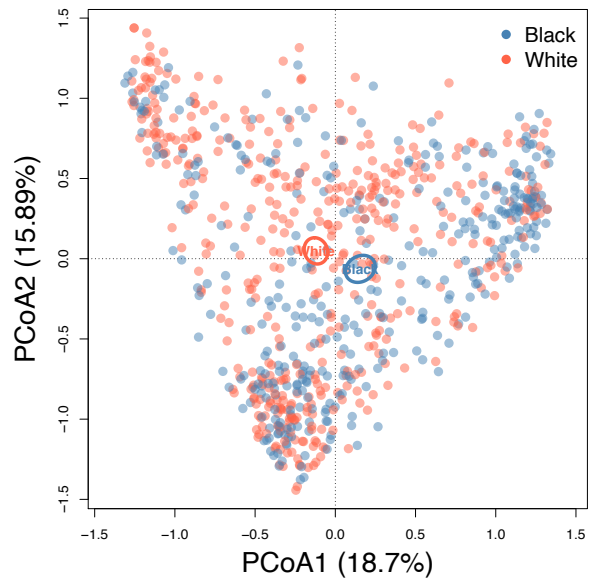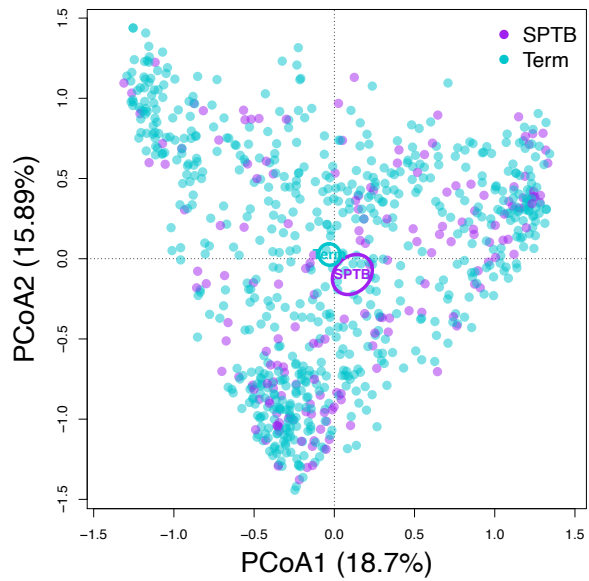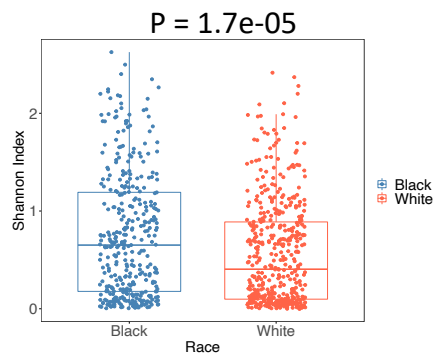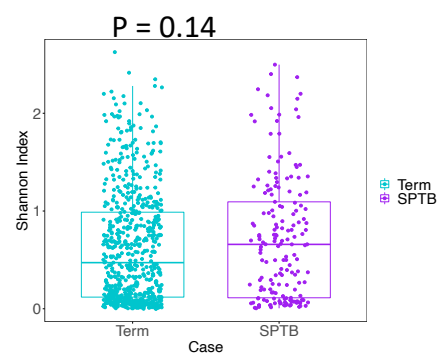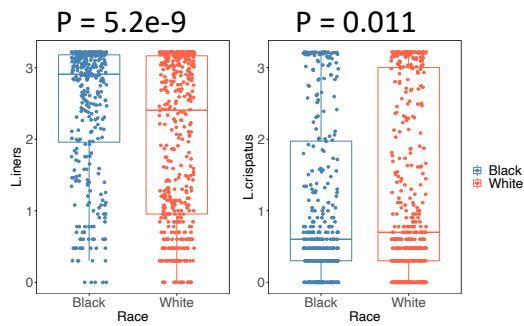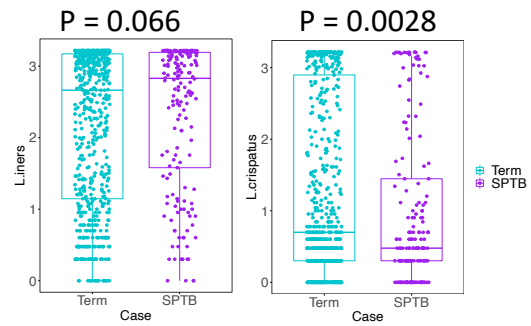

Supplement: FIG S6 [file msystems.00017-22-s0010.pdf]
