# Supplementary material for: Race, the Vaginal Microbiome, and Spontaneous Preterm Birth
Source: mSystems. 2022 May 18;7(3):e00017-22. doi: 10.1128/msystems.00017-22 (PMC9238383; doi:10.1128/msystems.00017-22)
Supplement: TABLE S1 [file msystems.00017-22-s0001.docx]

Table S1. PERMANOVA tests of microbiome and host factors.

|  | R2 | P | FDR |
| --- | --- | --- | --- |
| Race | 0.018 | 0.001 | 0.002 |
| Marital Status | 0.030 | 0.001 | 0.002 |
| Education | 0.044 | 0.001 | 0.002 |
| Age | 0.022 | 0.001 | 0.002 |
| Poverty level | 0.052 | 0.001 | 0.002 |
| Parity | 0.013 | 0.046 | 0.060 |
| Douching | 0.019 | 0.001 | 0.002 |
| Negative life events | 0.007 | 0.012 | 0.017 |
| Depression | 0.009 | 0.002 | 0.004 |
| SPTB | 0.004 | 0.010 | 0.016 |
