# Supplementary material for: Race, the Vaginal Microbiome, and Spontaneous Preterm Birth
Source: mSystems. 2022 May 18;7(3):e00017-22. doi: 10.1128/msystems.00017-22 (PMC9238383; doi:10.1128/msystems.00017-22)
Supplement: TABLE S2 [file msystems.00017-22-s0002.docx]

Table S2. Multivariable adjusted odds of spontaneous preterm birth associated with the covariates included in the models in Table2a.

|  |  | *L.crispatus/*  *L.iners/*  *Alpha-diversity* | White Race  (ref = Black) | Maternal Education  (ref = HS education or lower) | | | Maternal pre-pregnancy BMI (kg/m^2^) | Non-Smoking during pregnancy  (ref = smoking) |
| --- | --- | --- | --- | --- | --- | --- | --- | --- |
|  |  |  |  | Some College Education | College Degree | Post-graduate Degree |  |  |
| Overall | *SPTB ~ L.crispatus* | 0.81 (0.70, 0.94) | 0.86 (0.57,1.28) | 1.28 (0.79,2.07) | 1.27 (0.64,2.53) | 0.49 (0.2,1.2) | 0.99 (0.96,1.02) | 0.68 (0.42,1.08) |
|  | *SPTB ~ L.iners* | 1.06 (0.89, 1.27) | 0.84 (0.56,1.24) | 1.22 (0.76,1.97) | 1.19 (0.6,2.38) | 0.46 (0.19,1.13) | 0.99 (0.96,1.02) | 0.67 (0.42,1.07) |
|  | *SPTB ~ Alpha-diversity* | 1.20 (0.88, 1.64) | 0.84 (0.56,1.24) | 1.21 (0.75,1.94) | 1.16 (0.59,2.28) | 0.45 (0.19,1.09) | 0.99 (0.96,1.02) | 0.67 (0.42,1.07) |
| Black | *SPTB ~ L.crispatus* | 0.85 (0.68, 1.06) | NA | 1.78 (0.86,3.69) | 1.85 (0.62,5.52) | 0 (0,Inf) | 1 (0.97,1.04) | 0.84 (0.36,1.99) |
|  | *SPTB ~ L.iners* | 1.02 (0.76, 1.36) | NA | 1.72 (0.83,3.56) | 1.82 (0.61,5.39) | 0 (0,Inf) | 1 (0.97,1.04) | 0.88 (0.37,2.1) |
|  | *SPTB ~ Alpha-diversity* | 1.30 (0.84, 2.02) | NA | 1.7 (0.82,3.52) | 1.84 (0.62,5.46) | 0 (0,Inf) | 1 (0.97,1.04) | 0.9 (0.38,2.11) |
| White | *SPTB ~ L.crispatus* | 0.80 (0.65, 0.97) | NA | 0.94 (0.49,1.8) | 0.96 (0.39,2.34) | 0.4 (0.15,1.05) | 0.98 (0.94,1.02) | 0.64 (0.36,1.13) |
|  | *SPTB ~ L.iners* | 1.06 (0.84, 1.34) | NA | 0.89 (0.47,1.68) | 0.87 (0.35,2.13) | 0.38 (0.14,1) | 0.98 (0.94,1.01) | 0.62 (0.35,1.09) |
|  | *SPTB ~ Alpha-diversity* | 1.13 (0.73, 1.77) | NA | 0.88 (0.47,1.67) | 0.83 (0.35,2) | 0.37 (0.14,0.97) | 0.98 (0.94,1.01) | 0.61 (0.35,1.08) |
