# Supplementary material for: Race, the Vaginal Microbiome, and Spontaneous Preterm Birth
Source: mSystems. 2022 May 18;7(3):e00017-22. doi: 10.1128/msystems.00017-22 (PMC9238383; doi:10.1128/msystems.00017-22)
Supplement: TABLE S3 [file msystems.00017-22-s0003.docx]

Table S3. Associations of spontaneous preterm birth with *L. crispatus*, *L. iners* and alpha diversity in univariate models.

|  | Overall  OR (95% CI) | Black Women  OR (95% CI) | White Women  OR (95% CI) |
| --- | --- | --- | --- |
| *L. crispatus* (log10 relative abundance) | 0.78 (0.67,0.91) | 0.82 (0.66,1.03) | 0.76 (0.62,0.93) |
| *L. iners* (log10 relative abundance) | 1.16 (0.99,1.37) | 1.05 (0.8,1.37) | 1.2 (0.97,1.48) |
| Alpha diversity (Shannon index) | 1.29 (0.98,1.69) | 1.37 (0.94,1.99) | 1.12 (0.73,1.71) |
