# Supplementary material for: Race, the Vaginal Microbiome, and Spontaneous Preterm Birth
Source: mSystems. 2022 May 18;7(3):e00017-22. doi: 10.1128/msystems.00017-22 (PMC9238383; doi:10.1128/msystems.00017-22)
Supplement: FIG S3 [file msystems.00017-22-s0007.pdf]

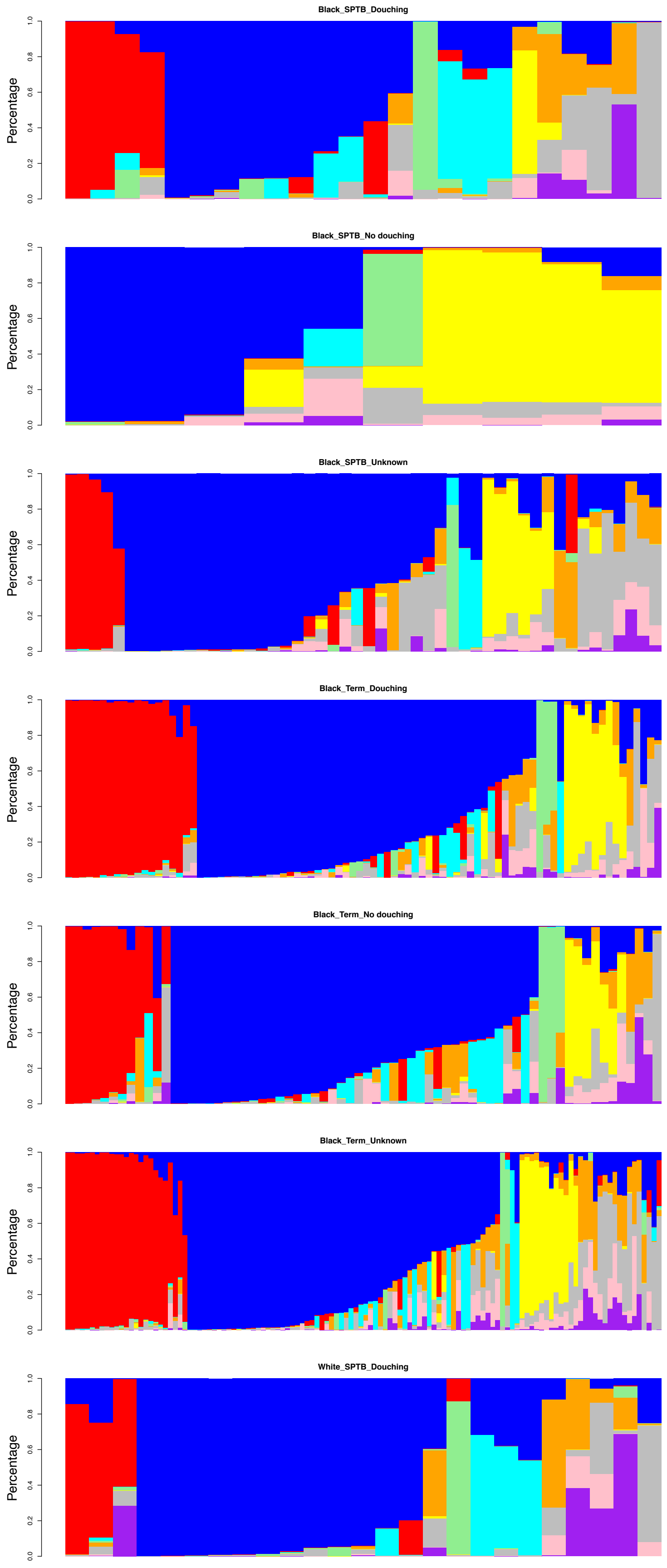
Black_SPTB_Douching
Percentage
0.0
0.2
0.4
0.6
0.8
1.0
Black_SPTB_No douching
Percentage
Black_SPTB_Unknown
Percentage
Black_Term_Douching
Percentage
Black_Term_No douching
Percentage
Black_Term_Unknown
Percentage
White_SPTB_Douching
Percentage

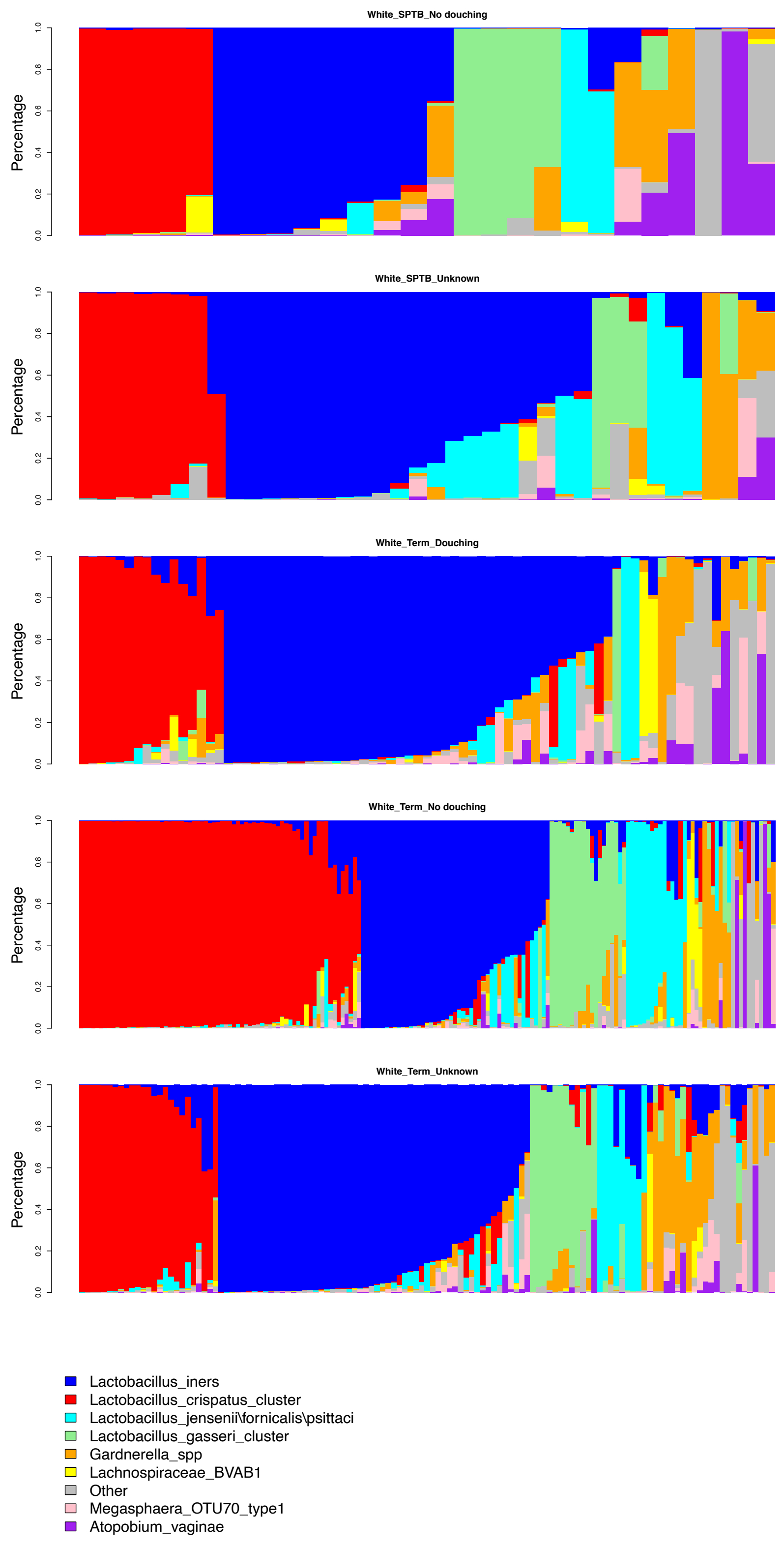
White_SPTB_No douching
White_SPTB_Unknown
White_Term_Douching
White_Term_No douching
White_Term_Unknown
Percentage
0.0
0.2
0.4
0.6
0.8
1.0
Lactobacillus_iners
Lactobacillus_crispatus_cluster
Lactobacillus_jensenii\fornicalis\psittaci
Lactobacillus_gasseri_cluster
Gardnerella_spp
Lachnospiraceae_BVAB1
Other
Megasphaera_OTU70_type1
Atopobium_vaginae
