# Supplementary material for: Race, the Vaginal Microbiome, and Spontaneous Preterm Birth
Source: mSystems. 2022 May 18;7(3):e00017-22. doi: 10.1128/msystems.00017-22 (PMC9238383; doi:10.1128/msystems.00017-22)
Supplement: TABLE S4 [file msystems.00017-22-s0004.docx]

Table S4. Characteristics of study participants with or without douching behavior within 12M before pregnancy (n = 489).

|  | **Self-reported douching within 12M before pregnancy** | | |
| --- | --- | --- | --- |
| **Maternal Characteristics** | **Yes**  **N=212**  **Mean (SD); N (%)** | **No**  **N=277**  **Mean (SD); N (%)** | **P-value** |
| Maternal Age (years)  Missing | 25.4 (5.8)  0 | 27.9 (6.7)  0 | 4.0e-05 |
| Maternal Education (years)  Missing | 12.3 (2.1)  0 | 14.6 (3.1)  0 | 5.0e-17 |
| Pre-pregnancy BMI (kg/m^2^)  Missing | 27.9 (7.9)  2 | 25.3 (7.0)  3 | 2.7e-05 |
| Smoking in the 2^nd^ Trimester (ever)  Ever  Never  Missing | 56 (26.4)  155 (73.1)  1 | 34 (12.3)  242 (87.4)  1 | 8.8e-05 |
| Parity  Nulliparous  Multiparous  Missing | 87 (41.0)  125 (59.0)  0 | 123 (44.4)  151 (54.5)  3 | 0.41 |
| Marital Status  Single/Separated/Divorced  Married  Missing | 131 (61.8)  81 (38.2)  0 | 94 (34.0)  183 (66.0)  0 | 1.2e-09 |
| Maternal Household % of Poverty level*  Missing | 162.6 (150.8)  8 | 335.4 (261.2)  11 | 7.8e-14 |
| CES-D Depression Symptoms | 19.3 (11.4)  48 | 14.4 (10)  34 | 1.6e-5 |
| Life Events Inventory, Number of Negative Life Events | 4.2 (3.1)  50 | 3.3 (3.2) 34 | 0.0012 |
